# Patterns and Predictors of Children's Musical Engagement From an Aotearoa NZ Longitudinal Cohort

**DOI:** 10.1002/snz2.70017

**Published:** 2026-02-22

**Authors:** Rebecca J. Evans, Daniel Yeom, Amy Tao, Ryan H. L. Ip, Bronya Dean

**Affiliations:** ^1^ Te Ipukarea Māori Research Institute Faculty of Design and Creative Technologies AUT Auckland New Zealand; ^2^ Melbourne School of Psychological Sciences Faculty of Medicine Dentistry and Health Sciences University of Melbourne Melbourne Australia; ^3^ Department of Mathematical Sciences Faculty of Design and Creative Technologies AUT Auckland New Zealand; ^4^ Te Kura Toi Tangata School of Education Division of Education University of Waikato Hamilton New Zealand

**Keywords:** childhood, cultural participation, extracurricular activities, longitudinal, music, parental singing, participation

## Abstract

In this article, we investigate patterns of children's musical engagement across childhood and early adolescence in Aotearoa New Zealand to identify key factors that predict sustained participation in musical activities outside formal education. Using data from multiple waves of the Growing Up in New Zealand longitudinal cohort study, children's musical engagement was assessed across five activity domains: singing, listening to music, watching videos (including music), music activity participation, and attending music events. Engagement in each domain was categorised into four participation levels: None, Short‐term, Repeated, and Sustained. Longitudinal patterns were assessed with Sankey plots, and ordinal regression was used to predict engagement levels from socioeconomic variables, including maternal education, household structure, household income, gender, ethnicity, disability status, area‐level deprivation, and rurality. Our results show that engagement patterns shifted with age, with family singing decreasing and individualised music listening and video watching increasing over time. Gender, ethnicity, and socioeconomic variables were key predictors of sustained engagement across activity domains. Children's musical engagement trajectories varied widely and were influenced by both individual characteristics and modifiable contextual factors. These findings highlight the need to consider equity of access and cultural relevance when supporting musical participation across childhood in New Zealand.

## 1 Introduction

Music has long been recognised for its significant role in child development, providing meaningful contexts for social interaction, emotional growth, cognitive development, and cultural learning (Hallam and Himonides 2022; Williams et al. 2015). Children engage with music in diverse ways through informal and formal musical experiences in both home and educational contexts (Creech et al. 2020). Understanding the patterns and factors that influence children's musical engagement is therefore essential for realising music's potential to support thriving childhoods.

Of the many factors that shape subsequent and lifelong engagement with music, family and home environments may be especially crucial. The familial environment is where most children first experience music (Mendoza and Fausey 2021; Lerma‐Arregoćes and Pérez‐Moreno 2023). In fact, infants can experience music before birth (Partanen et al. 2013), and evidence from acoustic analyses of proto‐conversations and musical games in infancy points to an inborn communicative musicality as being a core process in the human mind (Trevarthen 1999). From an early age, children are driven to engage with music, and over time, they exercise greater agency in selecting and engaging with music. This increased agency is scaffolded by both family preferences and the availability of resources that support music‐making (Lamont 2008). Shared musical activities—such as singing, dancing, and listening to music as a family—provide children with opportunities to engage with music, which may increase the likelihood of sustained engagement through adulthood (Theorell et al. 2015; Yeom et al. 2024). Children are now also growing up in increasingly digital and globally connected environments, meaning that the way in which they engage with music is being fundamentally reshaped by access to devices and the internet (Born 2022). Put simply, enriched early family environments are likely to foster sustained engagement, which will likely have downstream consequences for development as well.

Children's initial experiences within their families also foster enculturation, where they learn their culture's values, social norms and musical traditions (Kim 2007; Campbell 2011). Equally, a child's ethnic and cultural background contributes to shaping their musical engagement, as families engage in musical practices that reflect their ethnic and cultural traditions and values (Johnson‐Green and Custodero 2002; Sulkin and Brodsky 2021). These cultural practices not only mediate early musical engagement but are also instrumental in developing a sense of identity and belonging (Campbell 2011). To use singing as an example, sociocultural beliefs about singing (such as whether it is ’masculine’ for boys to sing) influence how likely children are to engage in it, which is likely to shape how their singing ability develops through to adulthood (Yeom et al. 2024). Cultures where singing is normative behaviour, in contrast, may be more likely to foster broader, continued engagement (Shilton et al. 2023).

Music engagement, family environments, and ethnic and cultural backgrounds are also likely to be shaped by broader socioeconomic and demographic factors. Socioeconomic status, which captures parental education, wealth and access to opportunities (Bradley and Corwyn 2002), likely plays a significant role in music engagement through determining access to music‐related resources (Hartas 2011). Families from higher socioeconomic backgrounds typically have greater access to resources for enrichment, which in turn promotes learning and engagement (Crosnoe et al. 2010). Evidence from Australia and the US points to significant socioeconomic influences on music engagement. Children from lower socioeconomic backgrounds are the most likely to report wanting to learn an instrument (McPherson et al. 2015), but the least likely to be enrolled in music classes in school (Elpus and Abril 2019; McPherson et al. 2015). The extent to which similar socioeconomic disparities are observed for other forms of musical engagement, particularly across the general population, remain largely unknown (though see Okely et al. 2021, 2022, for recent investigations).

Despite the growing body of research on musical engagement, important gaps remain. Many studies to date have relied on cross‐sectional data (data measured at one point in time), precluding detailed examination about how engagement changes over time, especially over multiple stages of childhood. For example, some studies ask participants to retrospectively recall aspects of their music engagement (e.g. Theorell et al. 2015). In addition, most longitudinal studies examining what sustains engagement with music have primarily focused on predicting engagement in future music education within school contexts (Ilari 2020), or operationalising music engagement as years of practise in music (Okely et al. 2021, 2022). Less focus has been given to how engagement in ‘informal’ non‐instrumental musical behaviours, such as listening to music or singing, changes over time. A related issue is that ’engagement’ has typically been defined in binary terms—whether children participate or not—without accounting for different levels of involvement. Research on music engagement and its impacts on cognitive and neurological development suggests that continued engagement leads to greater long‐term effects compared to short‐term or transient engagement (Merrett et al. 2013; Stekić 2024). As such, assessing music engagement in a binary way can mask important nuances of short‐ and long‐term engagement and their differing developmental outcomes.

In this article, we address several of these gaps by examining longitudinal changes in children's music engagement in Aotearoa New Zealand outside of formal music education contexts. Within Aotearoa New Zealand is a cultural landscape rich with musical traditions, yet scholarship on children's musical experiences is still emerging. To date, investigations here have primarily examined music within early childhood education settings (e.g. Bodkin‐Allen 2009; Naughton and Lines 2013) and in music therapy contexts (e.g. Wylie and Foster‐Cohen 2013), leaving little understanding of children's everyday musical experiences within home and family (with some exceptions, see Dean 2021). To the best of our knowledge, outside of our team, there are no current large‐scale studies of music engagement or development in the Aotearoa New Zealand context (Evans et al. 2022).

Our research draws on data from the Growing Up in New Zealand (GUiNZ) longitudinal cohort study, currently New Zealand's largest contemporary study of children's development. To date, GUiNZ has followed over 6000 children and their families, from before birth through multiple life stages up to 12 years of age, to explore the diverse realities of growing up in 21st‐century Aotearoa. The study was designed to be representative of the national birth cohort, and captures information across key domains such as family, education, health and wellbeing, societal context, cultural identity, and other childhood measures, including measures of musical engagement. GUiNZ has primarily investigated the psychological and demographic trajectories of children across Aotearoa New Zealand (see Morton et al. 2012 for an overview of the study's broad aims). In this study, we conduct secondary analyses of relevant music‐related data from the wider GUiNZ project.

Aotearoa New Zealand is highly ethnically and culturally diverse (Stats NZ 2023), with rich and varied musical traditions. Indigenous Māori musical practises (including *kapa haka*, a traditional performing art involving song and dance) contain deep links to Māori culture, identity, *reo* (language), *whanaungatanga* (connections), *hauora* (wellbeing), and form a crucial part of the nation's collective cultural identity (Pihama et al. 2014). The country's rich cultural diversity has enabled a thriving music scene, and children are often exposed to multicultural activities from a young age through formal and informal settings. Therefore, the Aotearoa New Zealand context allows for an examination of patterns of multicultural music engagement over time.

We asked two key questions in the present study. First, how does engagement with music change over the first 12 years of life? Second, what demographic, familial, cultural and socioeconomic factors predict different levels of engagement over time?

## 2 Materials and Methods

### 2.1 Data and Study Sample

Participants were members of the GUiNZ cohort study, for which cohort profile and recruitment information have been described elsewhere in depth (Morton et al. 2012; Morton et al. 2014). Briefly, pregnant women residing in Auckland, Counties‐Manukau, or Waikato—regions selected for ethnic and socioeconomic diversity—were recruited between April 2009 and March 2010. The subsequent cohort represented roughly 11% of all New Zealand births during recruitment, with characteristics broadly generalisable to the national birth cohort at the time (Morton et al. 2014). In this study, we draw on data collected through multiple data collection waves (DCWs) of the GUiNZ study to investigate children's musical engagement over time. This includes responses collected across 6 developmental stages: 9 months, 2 years, 54 months, 72 months, 8 years, and 12 years of age.

To minimise potential data clustering and bias, we excluded participating mothers with multiple births (e.g., twins or triplets) and included only those who responded across all DCWs examined and for whom musical engagement data were available (as per each category, see below). In addition, missing values, duplicate records, and records with inconsistent responses were removed. The resulting analytic samples for each activity consisted of 3702 children (singing), 3983 children (listening to music), 4026 children (watching videos), 3889 children (participating in musical activities), and 4402 children (attending musical events).

### 2.2 Music Engagement Activities

At each DCW, participants in the GUiNZ cohort and their mothers completed a series of questionnaires, covering a range of demographic, socioeconomic, psychosocial, and developmental variables. We extracted relevant questions related to music across all available waves and identified five music engagement activities: singing (parental or family singing), listening to music, watching videos (including music videos), participating in musical activities, and attending music events. We note that the questions asked about each musical activity at each DCW differ; for example, at age 8, the questions relating to participation in activities also include arts and dance. This is primarily because the questions were adapted in response to the children's average ages at each DCW to be developmentally appropriate and was also due to the long‐term nature of the study. For a full description of the music questions asked and response options available at each DCW, see Supporting Table 1.

Based on participants’ responses across the DCWs, we derived an ordinal variable representing each participant's level of engagement with each music engagement activity. The four levels of engagement were no/none, short‐term, repeated, and sustained, in line with Tymoszuk et al. (2020). Broadly, we used these levels to capture engagement both within and across the DCWs in which they engaged with music. For example, someone who reported frequently singing over multiple waves was categorised as having sustained engagement with singing. For frequency‐based activities (singing, listening, and watching videos), ordinal responses (e.g., Never to Daily) were numerically coded and summed across waves. The summed scores were then coded into one of the four engagement levels. For binary activities (musical activities and events participation), Yes/No responses per wave were coded as 1 or 0 and similarly summed across waves to define an engagement level. In some waves, the response options were re‐categorised to align with other waves. Take participating in music activities as an example, in DCWs at ages 2 and 12, the response options were binary (Yes/ No). Yet, at age 8, there were six options. In recoding the options, we considered all options with a frequency less than or equal to “Once a month” as “No”. Full details are provided in Supporting Table 1. Detailed descriptions of the musical engagement activities and the definitions of the engagement levels can be found in Table 1.

**TABLE 1:** Descriptions of music engagement variables from the GUiNZ study.

Engagement activity	Definition	Waves collected	Response^a^	Engagement level
Singing	Being sung to by the mother, or singing together	9 months 54 months 8 years 12 years	Never, Weekly, Daily, coded as 0, 1, and 2, respectively.	Total = 0 (No) Total = 1‐2 (Short‐term) Total = 3‐5 (Repeated) Total = 6‐8 (Sustained)
Listening to music	Individual music listening on a device	8 years 12 years	Never, Monthly, Weekly, Daily, coded as 0, 1, 2, and 3, respectively.	Total = 0 (No) Total = 1‐2 (Short‐term) Total = 3‐4 (Repeated) Total = 5‐6 (Sustained)
Watching videos	Watching videos, including music videos (e.g. YouTube)	8 years 12 years	Never, Monthly, Weekly, Daily, coded as 0, 1, 2, and 3, respectively.	Total = 0 (No) Total = 1‐2 (Short‐term) Total = 3‐4 (Repeated) Total = 5‐6 (Sustained)
Participation in musical activities	Active participation and membership in choirs, bands, kapa haka groups and similar music‐related groups	2 years 8 years 12 years	Yes/No, coded as 0 and 1, respectively.	Total = 0 (No) Total = 1 (Short‐term) Total = 2 (Repeated) Total = 3 (Sustained)
Attending music events	Attending events such as cultural festivals, musicals, community music events	2 years 72 months 8 years	Yes/No, coded as 0 and 1, respectively.	Total = 0 (No) Total = 1 (Short‐term) Total = 2 (Repeated) Total = 3 (Sustained)

a Some of the responses were recoded to make them consistent across all collection waves. The “No” category in Participation in musical activities and Attending music events included respondents who participated in these activities at a low frequency, see Supplementary Table 1 for full details. Also, “Yes” in Participation in musical activities at 8 years included art and dance as well as musical activities.

### 2.3 Demographic and Socioeconomic Predictors

To examine predictors of music engagement levels, we used a series of demographic and socioeconomic variables. For a full description of demographic variables used, please see Supporting Table 2. Further details on how these measures were collected and derived are described in Paine et al. (2023).

#### 2.3.1 Maternal Education

Mothers reported their highest achieved qualification at the antenatal DCW. This was recoded into a categorical variable with three levels: low (either no qualification or secondary school), medium (a diploma, trade certification, or NCEA Level 5–6), and high (a Bachelor's degree or higher).

#### 2.3.2 Household Structure

Mothers answered a series of questions regarding who lives in the household, parenting arrangements, and family structures at 12 years. These responses were used to derive a variable with four levels: solo parent, two or more parents, parent/s and extended family, and living with parent/s and non‐kin (Evans et al. 2024).

#### 2.3.3 Household Income

Cohort families reported on their total annual household income with one of seven response options at 54 months. These options were recoded into a three‐level categorical variable: low (below $50,000), medium (between $50,001 and $100,000), and high income (above $100,000).

#### 2.3.4 Gender

At 12 years, participants self‐reported their gender identity. Responses to this question were categorised into three levels: cisgender boy, cisgender girl, and an ‘other’ gender‐diverse category to collectively capture transgender and non‐binary children, as well as those who reported they were unsure of their gender.

#### 2.3.5 Disability

At 12 years, participants self‐completed the Washington Group Short Set (WG‐SS) Scale, a validated six‐item measure of functional disabilities (Madans et al. 2011), to report their experiences of disability in six areas, such as walking or communicating. Responses on the WG‐SS were recoded into a binary Yes/No variable indicating whether a child self‐reported any experiences of functional disability.

#### 2.3.6 Deprivation

Created by Stats NZ, the New Zealand Deprivation Index (NZDep) is a neighbourhood‐level measure of socioeconomic deprivation based on the Census's Statistical Areas, and ranges from 1 (least deprived) to 10 (most deprived). GUiNZ families were assigned an NZDep score based on their primary address. This variable was then collapsed into five bands (1–2, 3–4, 5–6, 7–8, and 9–10), representing quintiles of area‐level deprivation. At the 12‐year DCW, NZDep 2018 was used.

#### 2.3.7 Rurality

The Urban Rural Indicator (UR2018) is a binary measure defined by Stats NZ that classifies statistical areas into urban or rural.

#### 2.3.8 Prioritised Ethnicity

At 12 years, participants reported all the ethnic groups they self‐identified with. Following these responses, a *prioritised* ethnicity variable was derived, whereby a participant was categorised under one ethnicity based on the following order: Māori, Pacific, Asian, ‘Other’, and European. For example, if a participant reported identifying as Māori, their prioritised ethnicity was categorised as such. ‘Other’ in this context covers ethnic identities not captured in the other categories (see Paine et al. 2023).

### 2.4 Statistical Analysis

**FIGURE 1:**
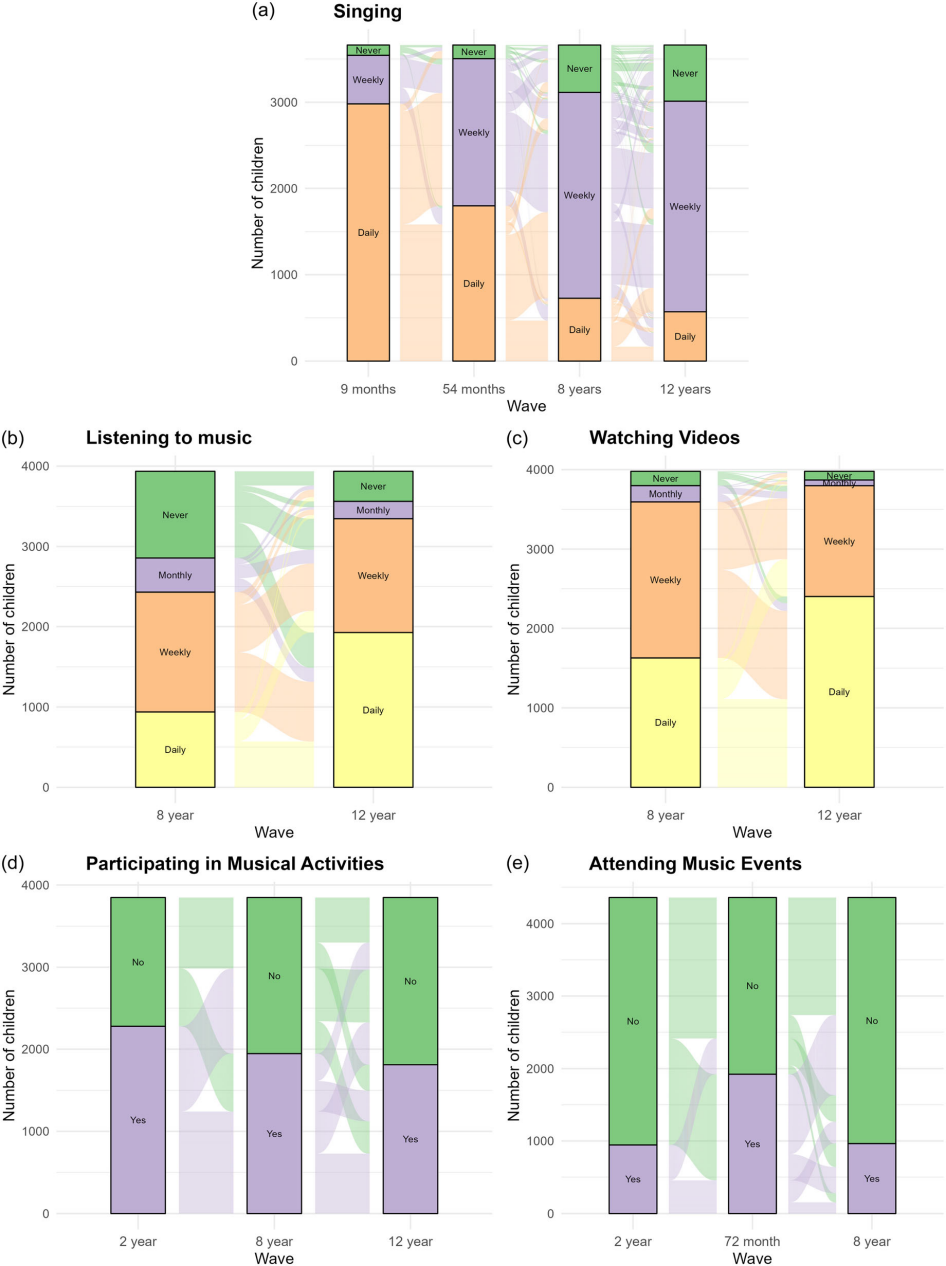
Sankey plot showing patterns of each music activity across waves. *Note:* (1) The music activities were collected at different waves, and none of them were collected at all five waves. (2) The original response options may be different across the waves. (3) In sub-plots (d) and (e), the recoded response “No” may include respondents who participated in music activities or events at a low frequency such as “once every 6 months” (see Supplementary Table 1). (4) In sub-plot (d), “Yes” at age 8 includes art and dance as well as music activities.

All analyses were conducted in R version 4.4.1 within the Amazon WorkSpaces platform provided by GUiNZ. Longitudinal patterns of musical engagement activities were visualised using sankey plots (Figure 1). For each musical engagement activity, the association between engagement levels and demographic and socioeconomic predictors was investigated using an ordinal regression model with cumulative logistic link functions (Agresti 2010). The model fitting was carried out using the *polr* function within the MASS package in R (Venables and Ripley 2002). The effects of the predictors are reported using adjusted odds ratios (AORs) with 95% confidence intervals (CIs). All models were checked for multicollinearity and statistical assumptions.

## 3 Results

### 3.1 Longitudinal Patterns of Music Engagement

Figure 1 shows how the GUiNZ cohort's frequency of musical engagement changes over DWCs across the five activities, prior to further categorisation into an overall engagement level. Singing frequency decreased over each timepoint; 81% of participants were sung to daily at 9 months old, but only 16% experienced daily singing at 12 years old. This largely corresponded to an increase in the proportion of children who were sung to weekly, as well as a modest rise in the proportion of children who were no longer being sung to. From 8 to 12 years, the frequency of both listening to music and watching videos (including music videos) increased. Specifically, the proportion of children who listened to music daily increased from 23% to 49%, while that for watching videos climbed from 41% to 60%. The proportion of cohort participants who were regularly engaged in musical activities dropped slightly from 2 to 8 years, then to 12 years (59%, 51%, and 47%, respectively). Finally, participation in music events increased from 2 years to 72 months, but subsequently decreased back to similar levels by 8 years (22%, 44%, and 22%, respectively).

### 3.2 Music Engagement Levels

The distribution of participants across engagement levels (No, Short‐term, Repeated, and Sustained) was different for each engagement type (Singing, Listening to Music, Watching Videos, Musical Activities, and Music Events). In the Singing category, almost all participants (96%) were categorised as having either Repeated or Sustained engagement. For musical events, the distribution was skewed to lower engagement, with most participants (78%) showing No or Short‐term engagement, and few (less than 5%) showing Sustained engagement. However, this may be due to the scoring method used in this work. Table 2 shows a summary of musical engagement levels in each of the five categories.

**TABLE 2:** Distribution of engagement levels across five musical activities (Singing, Listening to Music, Watching Videos, Musical Activities, and Music Events).

	Singing	Listening to music	Watching videos	Musical activities	Music events
No	160 (4.32%)^a^	180 (4.52%)	13 (0.32%)	554 (14.25%)	1639 (37.23%)
Short‐term		639 (16.04%)	158 (3.92%)	1305 (33.56%)	1814 (41.21%)
Repeated	1831 (49.46%)	1560 (39.17%)	2741 (68.08%)	1294 (33.27%)	792 (17.99%)
Sustained	1711 (46.22%)	1604 (40.27%)	1114 (27.67%)	736 (18.93%)	157 (3.57%)

a The two cells of ‘No’ and ‘Short‐term’ for Singing are combined as one of the two numbers is smaller than the reporting threshold.

### 3.3 Predictors of Stronger Music Engagement

**FIGURE 2:**
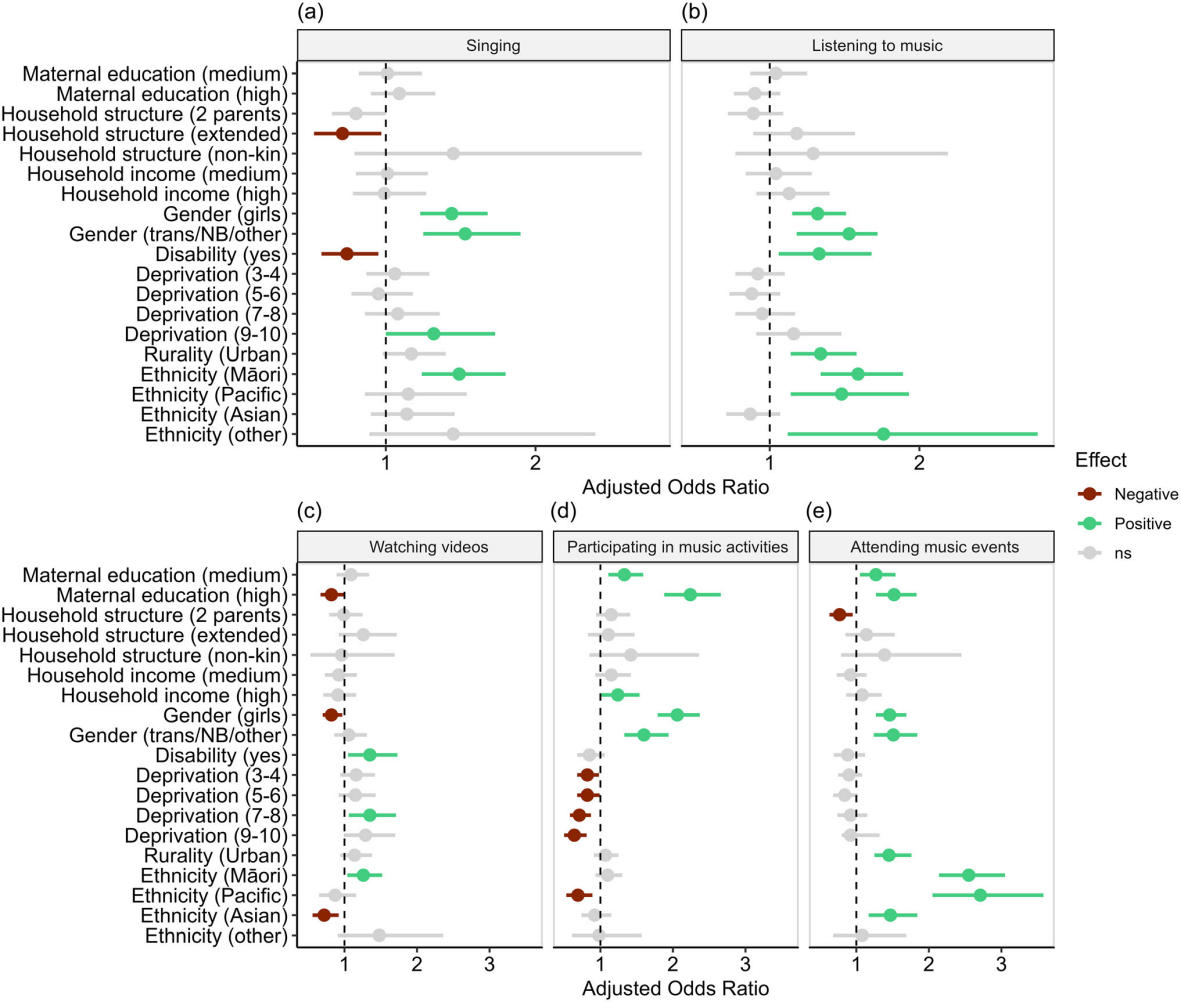
Effect size plots of predictors of the five types of musical engagement. *Note:* 95% confidence intervals are shown around each point estimate. The dashed vertical line indicates an adjusted odds ratio of 1. Effects with 95% confidence intervals crossing this vertical line indicate non-significant effects, and are shaded in grey. All highlighted effects are significant at *α* = 0.05; ns = non‐significant.

Figure 2 plots the effect sizes and corresponding 95% confidence intervals of the predictors for each of the five engagement activities (see Supporting Table 3 for full results). An AOR greater than one indicates a higher likelihood of having a stronger engagement level, compared to the reference (or baseline) category within the same predictor, assuming all other factors are held constant. Here, we focus on significant results, i.e., the factors that showed significantly different odds of having more sustained engagement with each form of musical engagement among the GUiNZ cohort participants.

#### 3.3.1 Singing

Girls (AOR = 1.44, *p* < 0.001) and gender diverse children (AOR = 1.53, *p* < 0.001) were more likely to experience sustained singing. Children in the highest level of deprivation (AOR = 1.32, *p* = 0.047) and children of Māori ethnicity (AOR = 1.49, *p* < 0.001) were also more likely to experience sustained singing across DCWs. Children living in extended family structures (AOR = 0.71, *p* = 0.032) and children with disabilities (AOR = 0.74, *p* = 0.017) were less likely to experience sustained maternal singing.

#### 3.3.2 Listening to Music

Girls (AOR = 1.32, *p* < 0.001), gender diverse children (AOR = 1.53, *p* < 0.001), children with disabilities (AOR = 1.33, *p *= 0.014), children in urban areas (AOR = 1.34, *p* < 0.001), Māori (AOR = 1.59, *p* < 0.001), Pasifika (AOR = 1.48, *p* = 0.003) and Other ethnic groups (AOR = 1.76, *p* = 0.015) were all more likely to sustain engagement with music listening. No variables predicted a lower likelihood of sustained engagement.

#### 3.3.3 Watching Videos

Children with disabilities (AOR = 1.35, *p* = 0.018), children from families in the 7–8 NZDep band (AOR = 1.35, *p* = 0.014), and Māori children (AOR = 1.26, *p* = 0.018) were more likely to have a higher level of engagement with watching videos. Children whose mothers had a high level of education (AOR = 0.82, *p* = 0.043), girls (AOR = 0.82, *p* = 0.017) and Asian children (AOR = 0.72, *p* = 0.009) were all less likely to sustain engagement in watching videos.

#### 3.3.4 Participation in Musical Activities

Children with more educated mothers (medium education AOR = 1.33, *p* = 0.002; high education AOR = 2.24, *p* < 0.001), children from high‐income households (AOR = 1.24, *p* = 0.05), girls (AOR = 2.06, *p* < 0.001) and gender diverse children (AOR = 1.60, *p* < 0.001) were more likely to sustain participation in music activities. In contrast, children at any level of deprivation (compared to little or no deprivation, NZDep band 1–2) were less likely to sustain participation in music activities, with the degree of deprivation further lowering the odds of staying engaged (AORs = 0.64‐0.82, all *p* < 0.05). Finally, Pacific children were less likely to sustain participation in music activities (AOR = 0.69, *p* = 0.004).

#### 3.3.5 Attending Music Events

Like activity participation, children with more educated mothers were more likely to sustain attendance at music events (medium education AOR = 1.27, *p* = 0.013; high education AOR = 1.52, *p* < 0.001). Girls (AOR = 1.46, *p *< 0.001), gender diverse children (AOR = 1.51, *p* = 0.031), children living in urban areas (AOR = 1.45, *p* < 0.001), Māori children (AOR = 2.55, *p* < 0.001), Pasifika (AOR = 2.71, *p* < 0.001) and Asian children (AOR = 1.47, *p* = 0.001) were also more likely to sustain engagement. In contrast, children living with two or more parents were less likely to have a higher level of engagement (AOR = 0.77, *p* = 0.016).

## 4 Discussion

### 4.1 Patterns of Children's Musical Engagement

We observed varying longitudinal patterns in five types of engagement with music over the first 12 years of life. Our results show that although most children engage with music in some form from early childhood through to adolescence, exactly how they do so changes as they age, with a notable “cross‐over” in singing and music listening frequency. As children entered adolescence, individualised forms of music engagement like listening increased, while maternal singing decreased. At 9 months, most mothers in the GUiNZ cohort sang songs or told stories daily to their children, a phenomenon also seen in many societies across the world (Fancourt and Perkins 2018; Yan et al. 2021; also see Evans et al. 2022). By 12 years old, however, most mothers sang with their children either once a week or less, showing a sharp decline in frequency. At the same time, music listening and watching videos—both forms of music consumption (Krause and Brown 2021)—increased from 8 to 12 years. These patterns are broadly consistent with findings from other countries, where parental singing decreases over time (Kreutz and Feldhaus 2018) while frequency of music listening peaks during adolescence and early adulthood (Bonneville‐Roussy et al. 2013; Williams et al. 2019). The increase of individual music consumption in adolescence is not surprising, given the importance of music for overall adolescent development (Miranda 2012), and the high importance young people place on music (North et al. 2000). These findings offer a novel contribution to longitudinal population research in Aotearoa New Zealand by demonstrating, with large‐scale cohort data, developmental patterns that align with international evidence and underscore the importance of tracking music engagement across childhood and adolescence at a population level.

While parents of young infants use singing as a parenting strategy (e.g., to calm or to structure their activities; see Dou and Cirelli 2025), parents change their strategies as children grow. However, when we examined longitudinal patterns grouped into levels of engagement, we saw that while the frequency of parental singing decreased, most families sang with repeated or sustained engagement over time. This suggests that families continue to value singing together even though it occurs less often. It is likely that the contexts for singing change, e.g., from parental singing to singing with the wider family and *whānau* (family), such as singing along to the radio in the car. Older children may also join in singing that is aimed at younger siblings, and siblings may sing amongst themselves. It is possible that the accessibility of recorded music through digital devices may have led to a decrease in parental singing (Yan et al. 2021), but evidence for this is mixed (Dou and Cirelli 2025).

Participation in musical activities became less frequent throughout childhood. Children were involved in musical groups or activities with reduced frequency over time, dropping from approximately two‐thirds at age 2 to one‐half by age 12. Importantly, at age 8, the question asked of participants also included art and dance activities, exact numbers at this age are likely lower for musical groups only. However, grouped levels of engagement were evenly distributed, with approximately one‐third of children engaging in short‐term, and one‐third in repeated engagement with musical activities. It is difficult to interpret this result, but it could simply indicate that while children participate less frequently overall, a similar proportion of children engage in some degree of involvement in activities (perhaps due to personal interest, *whānau* (family) support or access), as those who do not. Importantly, this result indicates that children are more likely to participate in some musical activity early in life.

Lastly, our results show low engagement with musical events. Just one‐fifth of children had been taken to a musical event in the past month at ages 2 and 8 years (with a small increase at 72 months), and most mothers reported either no or only short‐term involvement across multiple time points. This lack of engagement is somewhat surprising given the array of musical events on offer in Aotearoa New Zealand, particularly in Tāmaki Makaurau (Auckland), though to some extent it may also reflect our choice of scoring engagement frequency. Regardless, our findings indicate that families may face barriers to regularly attending music events. Events may not be promoted as family‐friendly; may be too expensive for families; or may be physically inaccessible, particularly for families living rurally or in smaller centres.

### 4.2 Predictors of Children's Musical Engagement

Sustained engagement in each music activity was predicted by a range of demographic and socioeconomic factors; however, several trends were evident. First, *tamariki* Māori (Māori children) showed higher sustained engagement in a variety of musical activities across time. While music is a human universal expressed in diverse forms (Hilton et al. 2022), cross‐cultural studies highlight the importance of cultural contexts when examining children's engagement with music. In Aotearoa New Zealand, very little research has focused on the early musical lives of children, particularly in relation to Māori and Pacific *whānau* (families). In Evans et al. (2022) earlier work, no differences were found between mothers of different ethnicities in their frequency of early parental singing, and in how often their young children listened to music, indicating the ubiquitous nature of music in the home during early childhood. In this article, towards mid–late childhood and adolescence, we found that *tamariki* Māori experienced higher rates of sustained maternal singing, listening to music, watching videos and attending musical events. These results suggest greater access to and/or value placed on sustained musical engagement in the home for *whānau* Māori.

Evans et al. (2023) have previously shown that many *tamariki* (children) engage in music through culturally‐embedded practises such as *kapa haka*. In *te ao* Māori (the Māori world), music is not only a recreational activity but a means of revitalising and maintaining language, strengthening *whanaungatanga* (relationships), identity, and connecting to culture, genealogy, history, and place (Kate 2024). Māori have a unique and enduring relationship with *waiata* (traditional Māori songs), *taonga pūoro* (traditional Māori musical instruments) and other musical forms, as documented in both studies of traditional Māori composers and contemporary music‐makers (e.g., Sheehan 2017; Royal 2012). In addition, Pacific children and children of other ethnicities were also more likely to report sustained listening to music, and both Pacific and Asian children were also more likely to report continued attendance at musical events, demonstrating greater involvement in these areas of musical engagement. Further research is needed to explore the context of this engagement.

Second, socioeconomic variables, including maternal education and area‐level deprivation, were significant predictors of many engagement activities after controlling for all other variables. These socioeconomic disparities were especially noticeable for enrichment activities, namely participation in musical activities and attendance at music events. Children from higher socioeconomic backgrounds—i.e. those with more educated mothers, and living in urban, and less deprived neighbourhoods—were more likely to sustain engagement with music activities and events. In short, access to music enrichment opportunities in Aotearoa, New Zealand is not equitable; children whose families had greater financial resources were also more likely to report sustained engagement in music activities. Our results align with evidence of socioeconomic disparities in music education (Elpus and Abril 2019; McPherson et al. 2015) and, importantly, suggest that these disparities extend to music engagement outside of formal education.

Third, girls and gender diverse children were more likely to consistently engage in all types of music compared to boys, except for watching videos. This deviates from an earlier finding with the same cohort that parental singing to 9‐month‐olds is not influenced by a child's gender (Evans et al. 2022). To some extent, these results may be explained by well‐documented evidence of the development of gendered stereotyping in music participation. For example, singing is stereotyped as “feminine”, and thus boys are less likely to sing as children (Yeom et al. 2024). Rather, boys in Western societies like Aotearoa New Zealand are often encouraged to take up more “masculine” activities like sports and are subsequently discouraged from music (Harrison 2007). These stereotypes may also explain why boys were also less likely to continue participating in music activities and attend music events. Contrary to our findings, gender differences have not been observed for music listening frequency in other research, including in adolescence (Williams et al. 2019). Therefore, it is likely that other factors shape music listening engagement.

Children reporting disabilities at age 12 were less likely to experience sustained parental singing, but more likely to sustain listening to music and watching videos. This may suggest more individualised forms of musical consumption for young people experiencing disabilities. In addition, raising a child with a disability has been associated with greater mental health concerns in parents (Chen et al. 2023), which may also impact the frequency of *whānau* (family) musical engagements, both interpersonal and individual. It is important to remember that we are not implying causality in this relationship: Further research is therefore needed to unpack the context surrounding these findings for *tamariki* (children) experiencing disability in relation to their musical engagement. Lastly, for children who live with extended family, we also found that their experiences of sustained maternal singing were lower. This makes sense if we consider that these children are likely cared for (and sung to) in part by the extended family living with them, so this finding may not be indicative of a true difference in experience.

Engaging with music has multiple benefits for overall development and wellbeing, particularly for cognitive and socioemotional development (Hallam and Himonides 2022; Lamont 2008; Stekić 2024; Williams et al. 2015). Our results broadly align with a previous finding that not all children in Aotearoa New Zealand have equal opportunities to engage with music experiences (Evans et al. 2022). Access to engagement opportunities during childhood is an important predictor of music engagement in later life, including uptake and continuation of music education (Crosnoe et al. 2010; McPherson et al. 2015; Theorell et al. 2015). The presence of disparities in informal contexts of music engagement suggests that multiple socioeconomic and sociocultural barriers may exist for children to experience the well‐established benefits of engaging with music. Whilst the formal schooling system is arguably set up to address disparities in opportunity, and music is a compulsory subject in the New Zealand curriculum, recent literature suggests that the reality of the provision and enactment of music education and extracurricular music opportunities through schools points to many children missing out (Wrathall 2024). It will be vital for both future research and policy to closely identify and examine barriers to formal and informal music participation, and how they can be minimised to foster opportunities to sustain lifelong engagement with music.

## 5 Limitations

There are some limitations to our study. First, the variables we used were self‐reported by participants or their mothers, which may have introduced bias. Additionally, the music engagement measures varied slightly across waves in terms of survey questions, response options and when they were measured. Our approach to categorise each engagement activity into no/short‐term/repeated/sustained engagement (based on Tymoszuk et al. 2020) aimed to mitigate these differences in data collection by deriving a standard measure of sustained engagement across activities and waves. Especially for participating in music activities and attending music events, caution must be applied when interpreting the results, as the recoded response ‘No’ at 72 months, 8 years and 12 years should be interpreted as ‘Not sufficiently regularly engaged’ (see Supporting Table 1 for details). Likewise, as we were conducting secondary data analyses from a broader longitudinal project spanning 12 years, there was missing data in all waves. Ideally, however, longitudinal studies of music should ask the same questions at the same timepoints throughout for greater consistency. This would allow for a stronger examination of changes in music engagement over time.

## 6 Conclusion

In this study, we show that children's engagement with music in Aotearoa New Zealand changes throughout the first twelve years of life. Most children's exposure to maternal singing decreased and they increasingly became independent consumers of music in multiple forms as they entered adolescence. The likelihood of a child sustaining engagement with each of the five musical activities was predicted by varying demographic and socioeconomic factors. We show that *tamariki* Māori (Māori children) demonstrated higher sustained engagement with music across childhood, reflecting *whānau* (family) practices where music is deeply valued as a way of nurturing identity, relationships, language, and cultural continuity. We also found evidence for a continuing gendered stereotyping of musical engagement for some activities. Finally, we found that not all children in Aotearoa New Zealand have equal opportunities to access music experiences, with specific evidence of socioeconomic disparities in sustained musical enrichment opportunities (activities and events).

Our findings point to the complex social and cultural dynamics that shape music engagement and raise important questions about who reaps the full benefits of engaging with music throughout childhood. We argue that a complete picture of children's musical development is incomplete without consideration of these nuanced dynamics and recommend that both future research and policy pay close attention to the various factors that enable or constrain access to music in the first years of life.

## Supporting Information

Additional supporting information can be found online in the Supporting Information section. **Supporting Table S1**: Full description of music questions, participants, variables and response options. **Supporting Table S2**: Full description of demographic variables. **Supporting Table S3**: Detailed ordinal regression results presenting the p‐values of the predictor variables based on likelihood ratio test, adjusted odds ratio (AOR), the corresponding 95% confidence interval (CI), and the p‐value based on z test for each level within the predictors.

## Funding

This study was supported by the New Zealand Government, Health Research Council, University of Auckland, and Auckland UniServices Limited.

## Ethics Statement

The *Growing Up in New Zealand* study was conducted according to the guidelines of the Declaration of Helsinki, ethics approval was granted by the NZ Ministry of Health Northern Y Regional Ethics Committee (NTY/08/06/055), and all participants gave their informed consent. Approval for this research was also obtained from the *Growing Up in New Zealand* study's Data Access Committee.

## Conflicts of Interest

No potential conflict of interest was reported by the author(s).

## Supporting information

Supplementary Material

## Data Availability

The data used in this manuscript were collected by the *Growing Up in New Zealand* study team. Researchers seeking access to this data may apply by submitting a data access application to the Data Access Committee at (dataaccess@growingup.co.nz).
