# Supplementary material for: Patterns and Predictors of Children's Musical Engagement From an Aotearoa NZ Longitudinal Cohort
Source: J R Soc N Z. 2026 Feb 22;56(1):e70017. doi: 10.1002/snz2.70017 (PMC12965002; doi:10.1002/snz2.70017)
Supplement: Supplementary file 1 — Supplementary Material [file SNZ2-56-e70017-s001.pdf]

Supplementary Table 1. Full description of music questions, participants, variables and response options.

	Question	Participants	DCW	Response options	Recoded responses
**Singing**	How often do you sing songs or tell stories to your baby [babies](do not include reading books)?	Child proxy by mother	9months	[1] Seldom or never<br>[2] Once a week<br>[3] Several times a week<br>[4] Once a day<br>[5] Several times a day	[0] **Never** – Never<br><br>[1] **Weekly** – Once a week, Several times a week<br><br>[2] **Daily –** Once a day, Several times a day
	How often do you sing songs or play music with {name}?		54 months		
	Using a scale of 1 to 5 where 1 is never or almost never and 5 is several times a day, overall how often do you do the following activities with {NAME}? - Singing a song, playing music, or doing some other musical activity with {NAME}?		8 years		
	Overall, how often do you do the following activities with {NAME}? - Sing, play music, or listen to music together		12 years	[0] Seldom or never<br>[1] Once a week<br>[2] Several times a week<br>[3] Once a day<br>[4] Several times a day	
**Listening to music**	How often do you do any of the following things using a screen or device, not including the time you are at school? - Listen to music	Child	8 years	[1] Every day<br>[2] Several times a week<br>[3] About once a week<br>[4] About once a month<br>[5] Hardly ever/never	[0] **Never** – Never<br><br>[1] **Monthly** – About once a month<br><br>[2] **Weekly** – About once a week, Several times a week<br><br>[3] **Daily** – Every day, Several times a day
	How often do you do these things using a screen device, not including the time you are at school? - Listening to music		12 years	[1] Several times a day<br>[2] Every day<br>[3] Several times a week<br>[4] About once a week<br>[5] About once a month<br>[6] Hardly ever/never	
**Watching videos**	How often do you do any of the following things using a screen or device, not including the time you are at school? - Watch TV or movies or videos (e.g. Netflix, Lightbox, TVNZ On Demand, YouTube and music videos)	Child	8 years	[1] Every day<br>[2] Several times a week<br>[3] About once a week<br>[4] About once a month<br>[5] Hardly ever/never	[0] **Never** – Never<br><br>[1] **Monthly** – About once a month<br><br>[2] **Weekly** – About once a week, Several times a week<br><br>[3] **Daily** – Every day, Several times a day
	How often do you do these things using a screen device, not including the time you are at school? - Watching TV, movies or videos (e.g. Netflix, Lightbox, Neon, AppleTV, YouTube, music videos)		12 years	[1] Several times a day<br>[2] Every day<br>[3] Several times a week<br>[4] About once a week<br>[5] About once a month<br>[6] Hardly ever/never	
**Participating in music activities**	Which of the these activities [child] has done or places [child] has been at any time since [he/she] was born: music groups?	Child proxy by mother	2 years	[0] No<br>[1] Yes	[0] **No** – No, Once per month, Once every 6 months, Once a year, Never

	Over the past 12 months, thinking about a normal week, how often has {NAME} participated in the following extracurricular activities? - Art, music, or dance, lessons, practice and performances (e.g. piano, dance, choir, drama, kapa haka)		8 years	[1] More than once per week<br>[2] Once per week<br>[3] Once per month<br>[4] Once every 6 months<br>[5] Once a year<br>[6] Never	[1] **Yes** – Yes, More than once per week, Once per week
	Thinking about the past year, which of the following activities do you do or have you done regularly (about once a week)? Music - 1. Waiata/choir; 2. Orchestra; 3. Kapa haka; 4. Jazz/blues band; 5. Traditional group; 6. Brass/concert band; 7. Guitar or ukelele group; 8. Instrument lessons; 9. Rock band/other band; 97. Other; 96. None of these	Child	12 years	[0] No<br>[1] Yes	
**Attending music events**	Which of the these activities [child] has done or places [child] has been at any time since [he/she] was born: Pasifika festival? - Pasifika festival, Polyfest, marae event, or concert/play/live show	Child proxy by mother	2 years	[0] No<br>[1] Yes	[0] **No** – Not since born<br><br>[1] **Yes** – At least once since born
	In the past month, has your Growing Up in New Zealand study child/children done any of these things with you or another family member? Gone to a concert, play, museum, art gallery		72 months	[1] In the past week<br>[2] In the past fortnight<br>[3] In the past month<br>[4] Not in the past month	[0] **No** – Not in the past month<br><br>[1] **Yes** – In the past week, In the past fortnight, In the past month
	Over the past 12 months, how often has {NAME} been to any of the following places or events? - Play, musical, dance, concert, circus or other live show		8 years	[1] More than once per week<br>[2] Once per week<br>[3] Once per month<br>[4] Once every 6 months<br>[5] Once a year<br>[6] Never	[0] **No** – Once every 6 month, Once a year, Never<br><br>[1] **Yes** – More than once per week, Once per week, Once per month

Supplementary Table 2. Full description of demographic variables.

Demographic Variable	Original Grouping	n	% of cohort	Recoded Grouping (Recoded Label)	n recoded	% of cohort recoded
**Mother's Highest Education Levels**	No sec School Qualification	491	7%	Low education level (1) †	2118	31%
	Sec school/NCEA 1-4	1627	24%			
	Diploma/Trade cert/NCEA 5-6	2082	31%	Medium education level (2)	2082	31%
	Bachelor's degree	1539	23%	High education level (3)	2603	38%
	Higher degree	1064	16%			
**Household Structure**	Sole parent	654	13%	(1) †		
	Two or more parents	3623	73%	(2)		
	Parent(s) living with extended family	577	12%	(3)		
	Parent(s) living with non-kin	100	2%	(4)		
**Household income**	<=20K	87	2%	Low income (1) †	974	18%
	>20K and <=30K	203	4%			
	>30K and <=50K	684	13%			
	>50K and <=70K	816	15%	Medium income (2)	1988	37%
	>70K and <=100K	1172	22%			
	>100K and <=150K	1333	25%	High income (3)	2445	45%
	>150K	1112	21%			
**Child's gender**	Boy/Mostly Boy	2110	46%	Boys (1) †		
	Girl/Mostly Girl	1738	38%	Girls (2)		
	Transgender/Non-binary/Unsure	746	16%	Gender diverse (3)		
Prioritised ethnicity	European	2320	52%	European (1) †	2320	52%
	Māori	1004	22%	Māori (2)	1004	22%
	Pacific	504	11%	Pacific (3)	504	11%
	Asian	571	13%	Asian (4)	571	13%
	MELAA	58	1%	Other (5)	97	2%
	Other	39	1%			
Disability	No disability/difficulty identified	4125	90%	No disability (1) †		
	Disability/difficulty identified	449	10%	Disability (2)		
NZDep	NZDep 1-2	1073	24%	(1) †		
	NZDep 3-4	998	22%	(2)		
	NZDep 5-6	869	19%	(3)		
	NZDep 7-8	736	16%	(4)		
	NZDep 9-10	793	18%	(5)		
Rurality	Urban	3671	82%	(1) †		
	Rural	798	18%	(2)		

† Reference level

Supplementary Table 3. Detailed ordinal regression results presenting the p-values of the predictor variables based on likelihood ratio test, adjusted odds ratio (AOR), the corresponding 95% confidence interval (CI), and the p-value based on *z* test for each level within the predictors.

	Singing		Listening to music		Watching Videos		Music Activities		Musical Events	
	**AOR (95% CI)**	**p**	**AOR (95% CI)**	**p**	**AOR (95% CI)**	**p**	**AOR (95% CI)**	**p**	**AOR (95% CI)**	**p**
**Maternal Education**		**0.555**		**0.146**		**0.004**		**<.001**		**<.001**
Low	Ref†		Ref		Ref		Ref		Ref	
Medium	1.01 (0.82,1.24)	0.933	1.04 (0.87,1.25)	0.648	1.09 (0.89,1.34)	0.392	1.33 (1.11,1.59)	0.002	1.27 (1.05,1.54)	0.013
High	1.09 (0.90,1.33)	0.373	0.90 (0.76,1.07)	0.227	0.82 (0.67,0.99)	0.043	2.24 (1.88,2.66)	<.001	1.52 (1.27,1.83)	<.001
**Household Structure**		**0.029**		**0.036**		**0.315**		**0.411**		**0.001**
Solo	Ref		Ref		Ref		Ref		Ref	
2 parents	0.8 (0.64,1.00)	0.056	0.89 (0.72,1.09)	0.249	0.99 (0.79,1.25)	0.935	1.15 (0.94,1.41)	0.165	0.77 (0.63,0.95)	0.016
Extended	0.71 (0.52,0.97)	0.032	1.18 (0.89,1.57)	0.257	1.26 (0.92,1.72)	0.156	1.11 (0.83,1.47)	0.480	1.14 (0.85,1.53)	0.387
Non-kin	1.45 (0.79,2.71)	0.239	1.29 (0.77,2.19)	0.337	0.96 (0.53,1.69)	0.885	1.42 (0.85,2.36)	0.176	1.39 (0.79,2.45)	0.250
**Household Income**		**0.976**		**0.362**		**0.737**		**0.139**		**0.103**
Low	Ref		Ref		Ref		Ref		Ref	
Medium	1.01 (0.80,1.28)	0.917	1.04 (0.84,1.28)	0.743	0.92 (0.73,1.17)	0.541	1.15 (0.93,1.42)	0.186	0.92 (0.73,1.14)	0.439
High	0.99 (0.78,1.27)	0.966	1.13 (0.91,1.40)	0.255	0.91 (0.71,1.16)	0.437	2.24 (1.00,1.54)	0.050	1.08 (0.86,1.35)	0.514
**Gender**		**<.001**		**<.001**		**0.020**		**<.001**		**<.001**
Boys	Ref		Ref		Ref		Ref		Ref	
Girls	1.44 (1.23,1.68)	<.001	1.32 (1.15,1.51)	0.001	0.82 (0.70,0.97)	0.017	2.06 (1.79,2.37)	<.001	1.46 (1.27,1.69)	<.001
Diverse	1.53 (1.25,1.90)	<.001	1.53 (1.18,1.72)	0.000	1.06 (0.86,1.31)	0.562	1.60 (1.33,1.94)	<.001	1.51 (1.24,1.84)	<.001
**Disability**		**0.017**		**0.013**		**0.019**		**0.147**		**0.291**
No	Ref		Ref		Ref		Ref		Ref	
Yes	0.74 (0.57,0.95)	0.017	1.33 (1.06,1.68)	0.014	1.35 (1.05,1.73)	0.018	0.85 (0.68,1.06)	0.147	0.88 (0.69,1.12)	0.292
**NZ Dep**		**0.206**		**0.186**		**0.147**		**0.002**		**0.338**
1-2	Ref		Ref		Ref		Ref		Ref	
3-4	1.06 (0.87,1.29)	0.557	0.92 (0.77,1.10)	0.355	1.16 (0.94,1.42)	0.172	0.82 (0.68,1.06)	0.033	0.9 (0.75,1.08)	0.259
5-6	0.95 (0.77,1.18)	0.667	0.88 (0.73,1.07)	0.198	1.15 (0.92,1.43)	0.221	0.82 (0.68,0.99)	0.039	0.84 (0.68,1.02)	0.076
7-8	1.08 (0.86,1.36)	0.514	0.95 (0.77,1.17)	0.598	1.35 (1.06,1.71)	0.014	0.71 (0.58,0.87)	0.001	0.92 (0.74,1.15)	0.477
9-10	1.32 (1.00,1.73)	0.047	1.16 (0.91,1.48)	0.225	1.29 (0.99,1.70)	0.064	0.64 (0.50,0.81)	0.000	0.92 (0.80,1.32)	0.860
**Rurality**		**0.090**		**0.001**		**0.172**		**0.434**		**<.001**
Rural	Ref		Ref		Ref		Ref		Ref	
Urban	1.17 (0.98,1.40)	0.091	1.34 (1.14,1.58)	0.000	1.14 (0.94,1.38)	0.173	1.07 (0.91,1.25)	0.434	1.45 (1.25,1.76)	<.001
**Ethnicity**		**0.001**		**<.001**		**0.000**		**0.014**		**<.001**
European	Ref		Ref		Ref		Ref		Ref	
Māori	1.49 (1.24,1.80)	<.001	1.59 (1.34,1.89)	<.001	1.26 (1.04,1.52)	0.018	1.1 (0.93,1.30)	0.250	2.55 (2.14,3.05)	<.001
Pacific	1.15 (0.86,1.54)	0.345	1.48 (1.14,1.93)	0.003	0.87 (0.65,1.16)	0.347	0.69 (0.53,0.89)	0.004	2.71 (2.05,3.58)	<.001
Asian	1.14 (0.90,1.46)	0.278	0.87 (0.71,1.07)	0.184	0.72 (0.56,0.92)	0.009	0.92 (0.74,1.15)	0.452	1.47 (1.17,1.84)	0.001
Other	1.45 (0.89,2.40)	0.141	1.76 (1.12,2.79)	0.015	1.48 (0.91,2.36)	0.109	0.98 (0.61,1.57)	0.938	1.08 (0.68,1.69)	0.751

† Reference level
